# Preventing eye injuries in quarries

**Published:** 2015

**Authors:** Richard Wormald, Daksha Patel

**Affiliations:** Coordinating Editor: Cochrane Eyes and Vision Group (CEVG), London, UK; E-learning Director: International Centre for Eye Health, London School of Hygiene and Tropical Medicine, London, UK.

**Figure:**
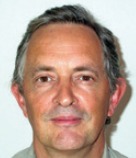
Richard Wormald

**Figure:**
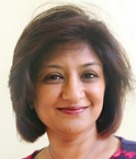
Daksha Patel

Eye injuries often occur in the workplace in low and middle-income countries, particularly in the construction, agricultural, mining, and manufacturing industries. Even if there are safety regulations in these industries, their enforcement is often unsatisfactory, and owners are not required to provide safety equipment.

In 2005, Christian Medical College in Vellore, India, conducted a pilot study in stone quarries in the area. At the start, they found that between 10% and 20% of workers had sustained injuries sufficiently severe for them to seek treatment (often costly) and that, of these injuries, 10% were sight threatening.

Plastic protective eyewear was then given to all workers after a single educational session (a health talk). Posters showing eye trauma due to quarrying were also displayed around the mine.

Regular use of protective eyewear was monitored by a health worker during surprise checks, and at three months 188/218 workers (86%) were regularly using them. A repeat slit lamp examination showed that the incidence of new eye injuries had reduced to 6% (13/218), and none were sight threatening.

The next challenge was to encourage sustained use of the eyewear, particularly as workers expressed their dissatisfaction, including: fogging and staining with sweat, a feeling of heaviness, and the development of scratches within two weeks, leading to difficulty with vision and requiring frequent replacement.

In order to answer the question ‘What is the evidence that educational interventions are effective in preventing ocular injuries?’ a Cochrane systematic review in 2009 assessed all available evidence and concluded that it was insufficient to answer the question, particularly in low-and middle income settings.

The Vellore group then carried out a follow-up randomised control trial (RCT) to assess the effectiveness of an educational strategy to encourage sustained compliance with the wearing of protective eyewear and to see whether this would reduce the incidence of eye injuries at three months and at six months.

In one arm of the RCT, a standard, single education session was provided, while in the other an enhanced education programme was provided over 11 sessions in 6 months. This included a standard talk, pre-recorded street plays and messages on the regular use of suitable protective eyewear, group motivational sessions, and individual counseling. In both arms, workers were given shatterproof, impact-resistant, heat-toughened protective eyewear with side shields.

Compared to standard education, the enhanced education had increased sustained compliance by 25%. The cumulative reduction in eye injuries over 6 months was greater in the enhanced education group (12%) than in the standard education group (7%) The effect of the intervention was limited by the small sample size and was not as large as in the pilot (which is often the case), but it was nevertheless a real effect.

This study demonstrated that:

- Protective eyewear designed to suit the harsh working conditions are accepted and welcomed by quarry workers
- Their regular use reduces the incidence of ocular trauma and prevents sight-threatening injuries
- Continued compliance with protective eyewear is improved by an enhanced educational programme that is sustained over longer periods than just one educational session.

Unfortunately, however, the eyewear used in the study was not easily available locally, and use of the eyewear (which was provided to quarry owners free of charge) was not sustained long after the study.

We recommend that researchers and eye health workers engaged in prevention of occupational eye injuries take the following action to ensure that evidence-guided service provision is established.

- Disseminate and discuss research findings with business owners (focusing on the economic benefit) and workers' unions (focusing on safety and rights in the workplace)
- Create links between the business (or quarry) owners and local protective eyewear providers – and encourage the local eyewear providers to offer bulk discounts
- Submit the findings to a local public health and occupational health authority.

